# Extracellular vesicle cross-talk in the bone marrow microenvironment: implications in multiple myeloma

**DOI:** 10.18632/oncotarget.7792

**Published:** 2016-02-29

**Authors:** Jinheng Wang, Sylvia Faict, Ken Maes, Elke De Bruyne, Els Van Valckenborgh, Rik Schots, Karin Vanderkerken, Eline Menu

**Affiliations:** ^1^ Department of Hematology and Immunology, Myeloma Center Brussels, Vrije Universiteit Brussels (VUB), Brussels, Belgium; ^2^ Department of Clinical Hematology, Universitair Ziekenhuis Brussel, Brussels, Belgium

**Keywords:** extracellular vesicle, exosome, multiple myeloma, bone marrow microenvironment, cross-talk

## Abstract

The bone marrow (BM) represents a complex microenvironment containing stromal cells, immune cells, osteoclasts, osteoblasts, and hematopoietic cells, which are crucial for the immune response, bone formation, and hematopoiesis. Apart from soluble factors and direct cell-cell contact, extracellular vesicles (EVs), including exosomes, were recently identified as a third mediator for cell communication. Solid evidence has already demonstrated the involvement of various BM-derived cells and soluble factors in the regulation of multiple biological processes whereas the EV-mediated message delivery system from the BM has just been explored in recent decades. These EVs not only perform physiological functions but can also play a role in cancer development, including in Multiple Myeloma (MM) which is a plasma cell malignancy predominantly localized in the BM. This review will therefore focus on the multiple functions of EVs derived from BM cells, the manipulation of the BM by cancer-derived EVs, and the role of BM EVs in MM progression.

## INTRODUCTION

The bone marrow (BM) is the source of blood cells and immune cells, and contains a complex environment composed of a cellular compartment, an extracellular matrix, and a liquid compartment [1]. The communication between BM-derived cells, mainly mediated by direct cell-to-cell contact, soluble molecules, and extracellular vesicles (EVs), maintains the physiological functions of the BM. Direct contact involves the interaction of a set of receptors and ligands on the cell membrane, leading to signal transduction and phenotypic changes in BM-derived cells, followed by the release of intercellular molecules [2, 3]. Soluble molecules, such as growth factors, cytokines, and chemokines, released from cells bind to the cognate receptors on the surface of target cells, leading to the activation or suppression of intracellular signaling pathways. EVs, a class of membranous vesicles, are secreted by various cell types into the extracellular microenvironment and mediate short- and long-range communication by stimulating target cells with receptors or ligands, transferring membrane receptors to target cells, delivering functional intracellular molecules, and inducing epigenetic changes in recipient cells [4, 5].

Based on the origin of the membrane, EVs can be classified into two primary types, termed exosomes and shedding vesicles [6]. Exosomes are nanometric membrane vesicles derived from late endosomes [7] and released through the fusion of multivesicular bodies (MVBs) with the plasma membrane [6, 8]. The formation of these exosomes starts in early endosomes, which are generated by invagination and endocytosis of the plasma membrane and its receptors. Intraluminal vesicles (ILVs) are generated inside the endosomes by inwards budding, and the early endosomes are then termed MVBs [9]. MVBs can either fuse with lysosomes or the trans-Golgi network, resulting in the degradation or recycling of the ILVs, or they can fuse with the plasma membrane, in which case the ILVs are released as exosomes [10]. Two major mechanisms are important in the formation and release of exosomes: the Endosomal Sorting Complex Required for Transport (ESCRT)-dependent mechanisms and the ESCRT-independent mechanisms [11]. The ESCRT exists of four different complexes, each involved in different steps of the formation of MVBs and the exosome release process [12]. Some of the ESCRT complexes used in the assembly and secretion of exosomes, such as ESCRT-III, are also important in the release of shedding vesicles [13]. Additionally, accessory proteins such as Alix, which is associated with ESCRT-III and promotes the intraluminal budding of vesicles inside the endosomes, are necessary for optimal biogenesis of exosomes [14]. Mechanisms independent from this ESCRT-complex involve lipids, tetraspanins or heat shock proteins. Ceramide has been shown to play an important role in exosome formation, loading and secretion, and inhibition of neutral sphingomyelinase can impair the secretion of exosomes [15, 16]. Shedding vesicles generally referred to as microparticles, ectosomes, microvesicles (MVs), exovesicles, and apoptotic blebs are secreted by direct budding or shedding from the plasma membrane [17]. Exosomes and shedding vesicles both contain enzymes, nucleic acids, transcription factors and proteins, and are enriched in certain lipid contents (sphingomyelin, cholesterol, glycerophospholipid and ceramide) [18]. Based on the EV types, different methods, including differential centrifugation, density gradient centrifugation, size exclusion chromatography, immune magnetic sorting or precipitation using easy-to-use precipitation solutions (eg. ExoQuick and Total Exosome Isolation) are commonly used. Due to the similar characteristics between some EV types (eg. microparticles and MVs) and the lack of standardized methods, the EV purity used in most of these studies is quite different, not unified and short of quality controls. Therefore there is an urgent need to establish standardized protocols to be able to interpret functional data correctly. Nevertheless, numerous functional studies have already been performed and will be discussed here.

In recent years, the close association of EVs with the immune response [3, 19], antigen presentation [17], tumor cell survival [20], cell migration [21–23], tumor cell invasion [24], cell differentiation [25], and angiogenesis [21, 23] has been demonstrated. As communicators, EVs are also involved in the regulation of the BM microenvironment and the communication between BM-derived cells and cells from other tissues. Recent evidence shows that tumor cells, such as breast cancer cells, melanoma cells, prostate cancer cells, and cholangiocarcinoma cells, from outside the BM can disturb the balance of the BM microenvironment and educate the BM-derived cells toward a pro-angiogenic and pro-metastatic phenotype through EV-mediated long-range communication [26–29]. Education of the BM microenvironment by tumor-derived EVs further support tumor growth, vasculogenesis, invasion, metastasis, and immune evasion. Thus, in this review we will provide a comprehensive overview of the biological functions of EVs, in particular exosomes, derived from normal or cancer cells in the BM microenvironment with a specific emphasis on the role in multiple myeloma (MM), a malignancy localized in the BM.

## BM CELL-DERIVED EVS

The cellular BM compartment consists of the hematopoietic cells which contain the intermediates of hematopoiesis and thrombopoiesis, plasma cells, and myeloid and lymphoid precursor cells, and the BM stroma, including adipocytes, BM stromal cells (BMSCs), mesenchymal stromal cells (MSC), osteoblasts, osteoclasts and endothelial cells. The EVs derived from the intermediates or precursor cells of hematopoiesis have not been investigated due to the instability of these not fully differentiated populations. Thus, studies mainly focused on the functions of EVs derived from BM stroma cells and fully differentiated hematopoietic cells and showed that each of these cell types produces EVs which affect homeostasis of the BM microenvironment and biological processes outside of the BM.

### MSC-derived exosomes

MSCs are multipotent stromal cells that are able to differentiate into a variety of cell types including osteoblasts, chondrocytes, myocytes, and adipocytes [30]. As a carrier of various cargo, the content in MSC-derived EVs has been investigated. Baglio *et a*l. have demonstrated that BM MSC-derived exosomes are highly enriched in tRNAs, representing > 35% of total small RNAs, while mature microRNAs (miRNA) account for only 2-5%, which differ from those in tissue specific MSC-derived exosomes [31]. In addition, a select pattern of miRNA important for metabolic processes, proliferation, differentiation and homing of stem cells are present in BM MSC-derived EVs [32]. Tomasoni *et al*. have demonstrated that BM MSC-derived exosomes horizontally transferred insulin-like growth factor-1 receptor (IGF-1R) mRNA to proximal tubular epithelial cells and increased their proliferation [33]. By using small RNA sequencing, proteomic, lipidomic, and metabolite assays, Vallabhaneni *et al*. have recently determined the total cargo of EVs derived from MSCs, and found several well-identified tumor supportive miRNAs (miRNA-21 and -34a) and proteins (PDGFR-β, TIMP-1, and TIMP-2), bioactive lipids (sphingomyelin and diacyl-glycerol), and metabolites (glutamic and lactic acid) [34].

Multiple functions of BM MSC-derived EVs have been revealed in normal biological processes and cancer progression. First, MSCs can modulate the immune system. Although this is largely mediated by paracrine factors, EVs secreted from these cells have also been reported to contribute to the regulation of the immune system. In a study by Favaro *et al*., BM MSC-derived EVs could be internalized by PBMCs (peripheral blood mononuclear cells) obtained from patients with a recent diagnosis of diabetes type I and suppressed their response to stimulation by suppressing the Th1 and Th17 response, upregulating regulatory T cells, and increasing IL-10, TGF-β and PGE2 [35]. In a different study, EVs could suppress the T cell response by inducing apoptosis of CD3^+^ and CD4^+^ cells in PBMCs (rather than inhibiting their proliferation) and by stimulating proliferation of regulatory T cells, leading to increased IL-10 [36]. In a study to reduce GvHD (graft *versus* host disease), MSC-derived exosomes were found to decrease the release of IL1-β, TNF-α, and IFN-γ from PBMCs and the secretion of TNF-α and IFN-γ from natural killer (NK) cells, further confirming their immunosuppressive activities [37]. Apart from immune effects, proangiogenic roles of MSC-derived EVs have been demonstrated as they can be taken up by endothelial cells and induce their proliferation, migration and tube formation [38, 39].

Effects of EVs derived from BM MSCs on cancer progression have also been studied in various cancer types. Ono *et al*. have demonstrated that MSC-derived exosomes promoted breast cancer cell dormancy in a metastatic niche by the transfer of miR-23b, and inhibited the proliferation and invasion of cancer cells [40]. An earlier study by Lim *et al*. has similarly determined that BMSC-derived exosomes favor breast cancer dormancy through transfer of miRNAs targeting CXCL12 and reducing cell proliferation [41]. Co-injection of MSC-derived exosomes with gastric carcinoma cells led to the promotion of both tumor incidence and growth in vivo [42]. MSC-derived EVs also favor the growth of breast cancer cells *in vivo*, indicating their tumor supportive function [34]. In contrast, Bruno *et al*. have found that EVs derived from MSCs inhibited the proliferation of three different tumor cell lines *in vitro* and *in vivo*, and these tumor cells tend to go into apoptosis or necrosis after treatment with EVs [43]. In view of the contrasting results in either tumor induction or suppression by MSCs and their EVs, it seems that the timing of injection is critical. EVs from MSCs co-injected with tumor cells seem to promote tumor growth, whereas they suppress tumor progression when they were administered after the tumor had been established [43]. Additionally, the source of the obtained MSCs seems to be of capital importance as shown by Del Fattore *et al*., who demonstrated that EVs from MSCs derived from either BM or the umbilical cord decreased proliferation and induced apoptosis of glioblastoma cells *in vitro*, while EVs from MSCs isolated from adipose tissue had opposite effects [44].

### Dendritic cell (DC)-derived EVs

DCs are antigen presenting cells originating from the myeloid lineage in the bone marrow and can exist in either a mature or immature stage. Immature DCs specialize in antigen capture, but cannot present them efficiently to T cells. They frequently induce immune tolerance in T cells due to low expression of MHC class I and class II, as well as costimulatory molecules such as CD80 and CD86. Activated mature DCs are among the most potent antigen presenting cells for activating T cells and carry higher levels of MHC and costimulatory molecules on their surface. Subsequently, these mature DCs efficiently induce the activation of the immune response upon stimulation [45]. Immature and mature DCs play important roles in initiating and shaping both the innate and adaptive immune responses, thereby regulating immune microenvironments in the BM or tumor [46].

Exosomes derived from DCs, also called dexosomes, have recently gained much attention in tumor vaccination studies as they express functional MHC class I and II and T cell co-stimulatory molecules which are essential for activating immunologic effector cells [47–50]. Exosomes secreted from mouse DCs were shown to induce antitumor effects through activating CD4^+^, CD8^+^ T cells, and invariant NKT cells [48, 51, 52]. Dexosomes loaded with antigenic protein or peptide, or exosomes derived from antigen-pulsed DCs promoted transgenic T cell proliferation *in vitro*, whereas only protein-loaded dexosomes induced T cell response and Th1-type memory *in vivo* in a B cell-dependent manner [53]. Protein-loaded dexosomes inhibited tumor growth in mice by inducing antitumor immunity in the assistance of proper activation of both CD4^+^ T and B cells [54]. Both murine and human dexosomes promoted NK cell proliferation and activation *in vivo* in an IL-15Rα- and NKG2D-dependent manner, resulting in tumor regression [49].

Because dexosomes reflect the phenotype of the parental DC at the time of isolation, dexosomes derived from mature DCs activate T cells more efficiently than those derived from immature DCs [55]. Indeed, dexosomes derived from immature DCs (imDex) have more immunosuppressive properties. They can suppress the development of myasthenia gravis in mice, by lowering both the proliferation of lymphocytes and production of antibodies. Lymphocytes from these treated mice exhibited lower expression of immune response factors such as IFNγ, TNFα and IL-6 [56]. Additionally, donor-derived imDex helped to induce immune tolerance in murine allograft models by inhibiting T cell activation, resulting in less rejection and longer survival of recipient mice [57–60]. Also, in sepsis, imDex containing milk fat globule epidermal growth factor-VIII (MFG-E8) enhanced macrophages-mediated phagocytosis of apoptotic cells and therefore decreased the inflammatory response [61, 62].

Some researchers have investigated whether changes in DCs can influence the dexosomes. Exosomes derived from DCs treated with IL-10 had immunosuppressive effects and could modulate the T cell response in an antigen specific and MHC class II dependent way [63, 64]. Exosomes from genetically modified DCs expressing Fas ligand (FasL), IL-4 or indoleamine 2,3-dioxygenase (IDO) were anti-inflammatory and immunosuppressive [65–67].

We can conclude that dexosomes can have conflicting functions, and it seems that the regulation of immune activation and tolerance mediated by dexosomes depends on the maturation stage of the originating DCs, the microenvironment where the exosome-DC interaction takes place, as well as the stimulation of DCs [68, 69].

### Other BM cell-derived EVs

Macrophages are derived from the monocyte lineage in the BM. EVs released from infected macrophages induced immune activation by activation of other naive macrophages, maturation of DCs, and activation of CD4^+^ and CD8^+^ T cells [70–72]. An earlier study by Singh *et al*. however indicated that exosomes from infected macrophages could transfer IFN-γ unresponsiveness to naive, uninfected macrophages and inhibit their activation [73]. Mast cells, derived from myeloid stem cells, secreted immunologically active exosomes which could induce B and T cell proliferation and promote the production of IL-2 and IFN-γ both *in vitro* and *in vivo*. These exosomes expressed multiple immunologically relevant molecules, such as MHC Class II, CD86, CD40, CD40L, LFA-1, ICAM-1, and CDC25 [74, 75]. Valadi *et al*. demonstrated that mast cell-derived exosomes contain various small RNAs including functional mRNAs and miRNAs and thus mediate the genetic exchange between cells [18]. In a model of osteoporosis, it was shown that EVs released from BM MSC-derived adipocytes could transfer adipogenic RNA to osteoblasts, resulting in the expression of adipocyte specific genes by osteoblasts, which ultimately led to increased marrow adiposity and bone loss [76].

EVs secreted by the cells in the BM are involved in various processes through affecting immune response, cell differentiation, angiogenesis, and cancer progression, as summarized in Table 1. However, as stated above, contradictory results frequently occur, which may be caused by the inconformity of target cell types, EV types, or the mouse model used in these studies. The establishment of unified investigation on the functions of EVs derived from the BM cells is needed to better understand the EV-mediated communications.

**Table 1:** The functions of EVs derived from BM cells

Source cell	Type of EVs	Target cell	Functions	Ref.
MSC	EVs	Treg, CD3^+^cell	Promote proliferation and apoptosis of Treg; induce CD3^+^ cell apoptosis	36
MSC	Exosomes	Immune cell	Induce immunosuppression	37
MSC	Microvesicles	PBMC	Decrease Th17 cells and increase Treg	35
MSC	Microparticles and exosomes	Proximal tubular epithelial cell	Promote cell proliferation	33
MSC	EVs	Endothelial cell	Promote tube formation and proliferation	38
MSC	40-150nm EVs	Breast cancer cell	Promote tumor growth *in vivo*	34
BMSC	Exosomes	Breast cancer cell	Reduce CXCL12 and decreased proliferation	41
MSC	Exosomes	Gastric cancer cells	Promote tumor growth *in vivo*	42
MSC	Microvesicles	Hepatoma, Kaposi's sarcoma, and ovarian tumor cell lines	Inhibit cell cycle progression; induce apoptosis; inhibit tumor growth *in vivo*	43
MSC	EVs	Glioblastoma cell line	Decrease cell proliferation, induced apoptosis	44
peptide-pulsed DC	Exosomes	T cell	Induce specific cytotoxic T lymphocytes *in vivo* and suppress tumor growth	48
DC	Exosomes	NK cell	Induce NK cell proliferation and activation *in vivo*	49
imDC	Exosomes	NK cell	NK cell activation	50
DC	Exosomes	CD4^+^ T cell	T cell activation	51
DC	Exosomes	iNKT cell	Activate iNKT cells and induce cancer-specific adaptive immune response	52
DC	Antigen-loaded exosomes	T cell	Induce specific transgenic T cell proliferation and response; Th1-type shift	53
DC	Antigen-loaded exosomes	CD8^+^T cell	Induce cytotoxic T cell response	54
imDC	Exosomes	Lymphocyte	Reduce proliferation	56
imDC	Exosomes	T cell	Suppress T cell response and increase IL-10 production	57
imDC	Exosomes	T cell	Immunosuppression	58
DC	Exosomes	T cell	Reduce T cell responses	59
imDC	Exosomes	T cell	Stimulate CD4^+^ T cells	61
imDC treated with IL-10	Exosomes	N/A	Suppress inflammation and autoimmune responses	63
DC over-expressing FasL	Exosomes	N/A	Induce antigen-specific immune responses *in vivo*	65
DC over-expressing IL-4	Exosomes	DC, macrophage, T cell	Suppress inflammatory response	66
Infected macrophages	50-300 nm EVs	Naive macrophage	Induce pro-inflammatory cytokines production	70
Infected macrophages	Exosomes	DC, T cell	Stimulate activation and maturation of DC; activate antigen-specific CD4^+^ and CD8^+^ T cells	71
Infected macrophages	Exosomes	Naive macrophage	Recruit and activate macrophage	72
Infected macrophage	Exosomes	Naive macrophage	Suppress IFN-γ mediated activation of naive macrophages	73
Mast cells	Exosomes	Spleen cell	Promote proliferation, IL-2 and IFN-γ production	74, 75
Adipocytes	Exosomes	Osteoblast	Induce the expression of adipocyte specific genes	76

EV, extracellular vesicle, MSC, mesenchymal stromal cells; Treg, regulatory T cells; BMSC, bone marrow stromal cells; DC, dendritic cells; iNKT cell: invariant natural killer T cell.

## EFFECT OF TUMOR-DERIVED EVS ON BM-DERIVED CELLS

Intercellular communication between cancer and normal cells in the tumor microenvironment plays a pivotal role in the development and progression of cancer. The various roles of tumor-derived EVs, especially exosomes, in the local communication and cancer progression within the tumor microenvironment have been well studied and discussed in recent years [77–79]. Allopatry of solid tumor cells and the BM makes direct contact impossible, whereas soluble factors and EVs can bridge the gap and facilitate long-range communication. Indeed, tumor-derived soluble factors promote the mobilization and accumulation of BM-derived cells, including endothelial progenitor cells, MSCs, and myeloid-derived suppressor cells (MDSCs), out of the BM into the circulation and tumor microenvironment [80–85]. Numerous studies have also shown that tumor-derived EVs may target BM-derived cells to modify the BM microenvironment which in turn supports the growth of tumor cells through modulating angiogenesis, tumor cell migration, immune response, drug resistance and metastasis.

It has been shown that microparticles from paclitaxel treated breast carcinoma cells contain osteopontin by which they induce BM-derived pro-angiogenic cell mobilization and colonization, leading to microvessel sprouting and increased angiogenesis, which ultimately accelerates tumor growth [86]. In a different study, the anti-VEGF-A antibody B20 reduced the level of VEGF-A enclosed in breast cancer cell-secreted microparticles and these vesicles were unable to activate endothelial cells and could not promote BM-derived pro-angiogenic cell colonization [87]. In addition, exosomes derived from hematological tumor cells such as chronic myelogenous leukemia cells and MM cells also contribute to angiogenesis [88–91]. Umezu *et al*. have reported that leukemia cell-derived exosomes transfer miR-92a to vein endothelial cells and enhance their migration and tube formation without affecting their growth [89]. Hypoxia, an important element in the cancer microenvironment, modulates the release and miRNA profile of exosomes from leukemia or myeloma cells, which finally leads to an enhancement of angiogenesis [88, 90].

Over the entire process of cancer development, cancer cells interact intimately with the immune system to favor immune evasion. Evasion of the immune response involves three major mechanisms: the selection of tumor variants resistant to immune effectors, immune-resistant changes in tumor cells, and formation of an immunosuppressive microenvironment [92]. The latter involves attracting immune suppressive cells from the BM through soluble factors or EVs. The most prominent of these immune suppressive cells is the MDSC. This cell type of myeloid origin interferes with T cell responses in a variety of pathologies, including cancer. It was demonstrated that tumor-derived exosomes could be taken up by BM MDSCs *in vivo* and these exosome-activated MDSCs not only suppressed T cell activation, they also enhanced tumor growth [93, 94]. In addition, tumor-derived exosomes can increase cytokine production by the MDSCs [28]. From a mechanistic point of view it has been reported that the STAT3-dependent immunosuppressive activity of mouse and human MDSCs is induced by membrane-associated Hsp70 on tumor-derived exosomes [95]. These findings emphasize the involvement of tumor-derived exosomes in immunosuppression, leading to an acceleration of tumor progression.

Tumor-derived EVs are also involved in the induction of various immunomodulatory effects through impacting BM-derived cells. Mammary carcinoma cell-derived EVs were found to contribute to the enhancement of the innate inflammatory response mediated by macrophages [96] while melanoma cell-derived exosomes could activate macrophages through the NF-κB pathway and alter their cytokine/chemokine profile [97]. In addition, these exosomes promoted the maturation of DCs, leading to an enhanced T cell proliferation [97]. In contrast, Yu *et al*. have shown that mammary tumor cell-derived exosomes inhibited differentiation of BM DCs and monocytes [94]. Finally, body fluid exosomes obtained from ovarian cancer patients could enhance the secretion of inflammatory factors, such as IL-1β, TNF-α, and IL-6, in monocytic cells through toll-like receptor signaling [98].

**Figure 1:**
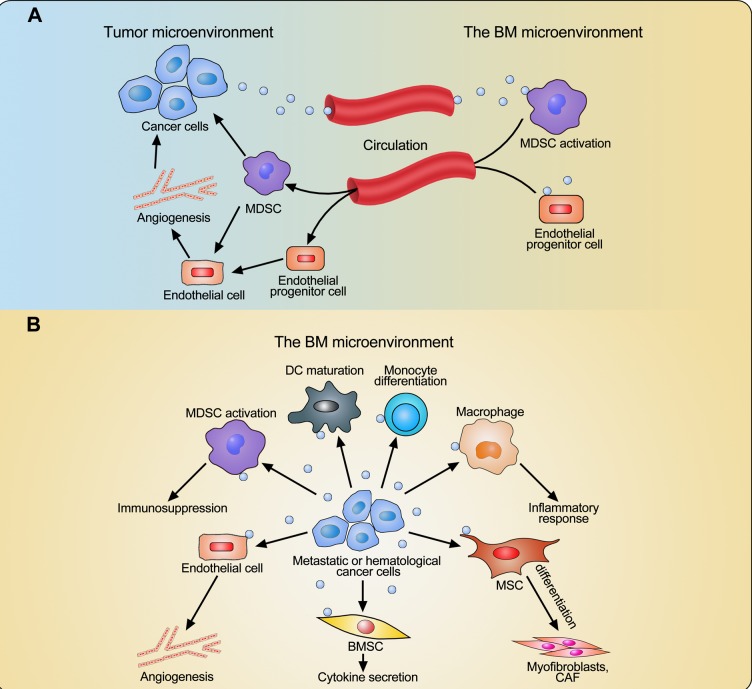
Interaction between cancer cells and BM-derived cells through extracellular vesicles (EVs) **A.** EVs derived from cancer cells outside of the BM induce the mobilization and localization of MDSCs and endothelial progenitor cells from the BM into the tumor microenvironment and therefore enhance angiogenesis and immunosuppression. **B.** EVs derived from metastatic or hematological cancer cells induce cell differentiation and maturation, inflammatory response, cytokine secretion, angiogenesis, and immunosuppression in the BM microenvironment through affecting BM-derived cells, including monocytes, dendritic cells (DCs), macrophages, mesenchymal stromal cells (MSCs), BM stromal cells (BMSCs), endothelial cells, and myeloid-derived suppressor cells (MDSCs). This educated BM microenvironment facilitates cancer cell growth.

In recent years, it has been found that EVs can mediate the crosstalk between malignant cells and BMSCs or MSCs. One study reported that BMSCs pulsed with hepatocellular carcinoma-derived exosomes had higher migratory capacities and strong antitumor activities [99]. However by contrast, Corrado *et al*. have shown protumoral effects of BMSCs treated with chronic myelogenous leukemia cell-derived exosomes as these BMSCs secreted more IL-8, promoting leukemia cell growth *in vitro* and *in vivo* [100]. In a different report, prostate cancer cell-derived exosomes could trigger MSC differentiation into myofibroblasts which ultimately promoted angiogenesis, tumor cell proliferation and invasion [101]. Similarly, Haga *et al*. have demonstrated that human cholangiocarcinoma cell-derived EVs induced fibroblastic differentiation in MSCs and these MSCs in turn facilitated tumor cell proliferation and migration [102]. Exosomes released by chronic lymphocytic leukemia cells induced a phenotype of cancer-associated fibroblasts in MSCs and these stromal cells showed enhanced proliferation, migration and secretion of cytokines, which could further favor tumor cell growth [103]. Furthermore, melanoma exosomes educated BM progenitor cells through the receptor tyrosine kinase MET and reprogrammed them towards a pro-angiogenic and pro-metastatic phenotype, which could support tumor growth and metastasis [26]. The complex communication between cancer cells and BM-derived cells through EVs is illustrated in Figure 1.

## EVS IN MM

MM is an often incurable plasma cell neoplasm, which accounts for about 10% of all hematological cancers and is therefore the second most common hematological malignancy [104, 105]. Malignant plasma cells home to and are predominantly localized in the BM, since this microenvironment is crucial for MM pathogenesis and progression. Homeostasis of the BM microenvironment is disrupted and modified by the presence of the MM cells, leading to angiogenesis, osteolysis, immune suppression and anemia. Recent research has demonstrated that EVs should also be considered as one of the mediators by which MM cells induce BM microenvironmental modifications, as well as the involvement of EVs in MM development and induction of drug resistance, which will be discussed below.

### EV as a biomarker in MM

Elevated levels of total EVs in the body fluids are found in cancer patients as compared to healthy donors and they are reported to be correlated with cancer stages [106, 107]. In MM, it has been shown that serum samples from 72 MM patients with immunoglobulin light chain amyloidosis contained a higher level of MVs and exosomes than healthy donors [108]. Caivano *et al*. have recently studied serum EVs in patients with various types of hematological neoplastic disorders and demonstrated similarly that the concentration of serum EVs is significantly elevated in MM patients [109]. In addition, higher level of serum EVs expressing the ectoenzyme CD38 [110], a marker of plasma cells and an often used MM-related antigen was found, implicating that these vesicles originate from MM cells [109]. Similarly, Benameur *et al*. have found an increase of the total circulating and BM-derived microparticle (MP) number in a mouse model at end stages of MM compared to controls. Phenotypic characterization showed that circulating MPs originated from platelets, leukocytes, endothelial cells, and erythrocytes, while the BM-derived MPs express CD138, another marker for plasma cells [111]. The amount of serum EVs in MM patients has been evaluated by Di Noto *et al*. through the measurement of their acetylcholinesterase activity. The highest EV amount was found in MM patients in contrast to healthy donors and patients with monoclonal gammopathy of undetermined significance (MGUS) [112]. Moreover, exosome secretion from MM cells was shown to be dramatically increased by heparanase whose expression is up-regulated in aggressive cancer cells including MM cells, implying that heparanase may be one of the factors causing elevated secretion of exosomes [113].

A recent study identified 158 differentially expressed circulating exosomal miRNAs, including let-7 family members, miR-17/92 and miR-99b/125a clusters, in MM compared to normal healthy controls and demonstrated that the circulating exosomal miRNA signature was an independent prognostic marker in MM [114]. Moreover, some of these miRNA correspond to those previously identified as differentially expressed between MM cells and normal PCs [115]. Another similar study demonstrated that specific exosomal miRNAs are differentially expressed between high and low-risk MM patient, further suggesting the potential of exosomal miRNA signatures as a prognostic marker [116]. All these findings suggest that both total serum and tumor antigen-specific EVs including exosomes may represent a novel biomarker in MM and can be used for MM prognosis; however, more studies containing a large number of clinic samples are still needed to further confirm the correlation between EVs and MM progression.

### Characterization of MM cell-derived EVs

The proteomic content of EVs derived from two human MM cell lines has been analyzed by Harshman *et al*. [117] and they identified 583 vesicular proteins from these vesicles, including antigen presenting molecules, adhesion molecules, membrane transport and fusion molecules, cytoskeletal proteins, pyruvate kinase, histones, and other bioactive proteins involved in multiple biological processes [117]. Enriched membrane molecules such as CD44, MHC class I, and bone marrow stromal antigen 2 (BST-2) were observed in MM cell-derived MVs as compared to MM cells [117]. Elevated expression of CD147 was also found in human MM cell line-derived MVs, as well as in MVs from MM BM plasma. Ig light chain-positive MVs from MM patients express higher levels of CD38, CD319, CD44, and CD9 than those from MGUS patients [118]. Moreover, it has been shown that above 80% of MVs derived from a human MM cell line express CD138 [119].

### Function of MM cell-derived EVs

MM cell-derived EVs have been shown to alter different processes in the BM, such as angiogenesis and osteolysis, to enhance MM growth. Angiogenesis is essential for tumor growth, invasion and metastasis as tumor cells require more nutrients and oxygen from blood vessels. In MM patients, increased angiogenesis has been demonstrated in the BM and this higher microvessel density is associated with poor prognosis [120–122]. Enhancement of angiogenesis is mainly mediated by the well-known pro-angiogenic factors such as vascular endothelial growth factor (VEGF), basic fibroblast growth factor (bFGF), and angiopoietin-1 secreted by the MM cells or stromal cells interacting with MM cells [123–128]. Two recent studies have demonstrated MVs or exosomes derived from MM cells as new inducers of angiogenesis [88, 119]. MVs secreted from a human MM cell line directly promoted the proliferation and capillary structure formation of umbilical vein cells, and induced angiogenesis *in vivo*. These EVs promoted the expression and secretion of VEGF in umbilical vein cells, which can further enhance angiogenesis [119]. Hypoxia-inducible factor (HIF)-1α is often overexpressed by MM cells due to the hypoxic BM environment, leading to increased secretion of proangiogenic cytokines [129, 130]. Umezu et al [88] found that MM cells under hypoxic conditions secreted more exosomes with increased levels of miR-135b which induced increased expression of HIF-1α in endothelial cells, leading to enhanced angiogenesis. Moreover, serum EVs obtained from MM patients clearly promoted the proliferation of human vascular endothelial cells as compared to those from healthy donors or MGUS patients [112]. A very recent paper has suggested that fibronectin on the surface of MM cell-derived exosomes binds to heparin sulfate on endothelial cells thereby mediating MM exosome-BM cell interactions [131].

In MM, increased osteoclastic activity together with suppressed osteoblastic activity is the main cause of osteolytic bone disease [132]. The differentiation and activation of osteoclasts are mainly mediated by osteoclast activating factors secreted by the MM cells or BMSCs in the BM [1, 132]. The increased number and activity of osteoclasts further enhance MM cell growth and survival through cell-cell contact and secretion of IL-6 and B-cell-activating factor [133–135]. A recent paper has demonstrated MM cell-derived exosomes as mediators of osteoclast formation and activation [136]. MM-derived exosomes supported both survival and migration of osteoclast precursors, and induced their differentiation to osteoclasts, as well as their bone resorption activity. Moreover, exosomes obtained from the plasma of MM patients exhibited the same functions in osteoclast differentiation [136].

Interestingly, EVs can also directly promote MM cell proliferation in an autocrine manner. Specifically, EVs derived from CD147-overexpressing MM cells or MM patients enhance MM cell proliferation, while the growth promotion of EVs from CD147-downregulated cells was attenuated, suggesting that CD147 is partially involved in EV-induced cell proliferation [118].

Tumor-derived exosomes generally contain tumor antigens and therefore have attracted much attention for their potential use as vaccines [137–139]. These exosomes still need the host DCs to present tumor antigens for the stimulation of antitumor immunity [140–142]. The antitumor response mediated by MM cell-derived exosomes was examined after engineering modifications. Exosomes released by MM cells engineered to express TNF-α induced a more efficient tumor antigen-specific CD8^+^ T cell response in mice and protected all the experimental mice from tumor growth [143]. Exosomes collected from MM cells overexpressing membrane-bound Hsp70 stimulated maturation of DCs and upregulated the presence of several membrane molecules such as la^d^, CD40, and CD80, as well as the secretion of inflammatory cytokines such as IL-1β, IL-12, IFN-γ, and TNF-α [144]. Moreover, these engineered MM cell-derived exosomes stimulated a type 1 CD4^+^ T cell response in mice and protected tumor-bearing mice from death through induction of strong CD8^+^ cytotoxic T lymphocyte (CTL) responses and NK cell-mediated antitumor immunity [144].

**Figure 2:**
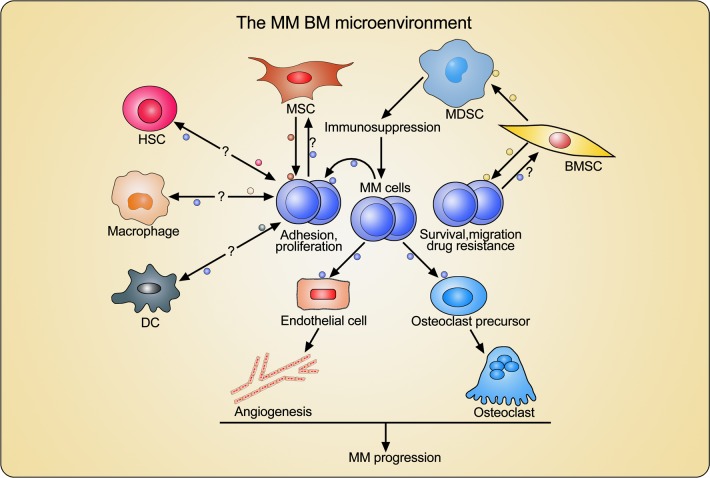
Crosstalk between MM cells and BM-derived cells through extracellular vesicles (EVs) MM cell-derived EVs modify the BM microenvironment through enhancing angiogenesis and promoting osteoclast differentiation and activation. BMSCs and MSC-derived EVs directly promote MM cell adhesion, proliferation, and survival, and induce drug resistance. BMSC-derived EVs indirectly facilitate MM progression through activating MDSCs and inducing immunosuppression. However, the effects of MM cell-derived EVs on MSC and BMSC, as well as the EVs-mediated interactions between MM cells and other important BM-derived cells such as hematopoietic stem cells (HSCs), macrophages, and dendritic cells (DCs), still need to be investigated.

Taken together, MM cell-derived EVs seem to enhance angiogenesis and osteoclastic activity in the BM, hereby establishing a favorable microenvironment and ultimately favoring MM progression (Figure 2).

### Function of stromal cell-derived EVs

BMSCs play crucial roles in MM progression and induction of drug resistance through cell-cell contact and secretion of cytokines [1, 145, 146]. MM cells adhere to BMSCs, stimulating the latter to secrete more soluble factors which mediate MM cell growth, survival, migration, drug resistance, and BM angiogenesis [145, 147–151]. Roccaro *et al*. and our group have demonstrated exosomes as novel communicators and regulators in the interactions between MM cells and stromal cells [152, 153].

The content of BM-MSC-derived exosomes from MM patients was analyzed by miRNA and protein arrays, showing a lower level of the tumor suppressor miR-15a and higher levels of oncogenic proteins, cytokines, and adhesion molecules compared to healthy BM-MSCs [152]. The inability of exosomal miR-15a transfer from tumoral BM-MSCs to MM cells partially contributed to the increased tumor burden in a murine MM model, since low levels of miR-15a were unable to keep MM cell proliferation [152]. Many cytokines including IL-1ra, interferon-inducible protein 10, monocyte chemoattractant protein 1 (MCP-1), macrophage inflammatory protein-1α (MIP-1α), MIP-1β, and stromal cell-derived factor 1 (SDF-1) were detected by our group in murine MM BMSC-derived exosomes [153]. We found that these exosomes carrying functional miRNA or proteins could be taken up by MM cells and induce their proliferation, survival, and migration, as well as drug resistance to bortezomib, a widely used and efficient clinical drug for MM treatment [153]. These findings highlight the roles of stromal cell-derived exosomes in supporting MM pathogenesis and progression. Additionally we identified an indirect approach to favor MM progression by stromal cell-secreted exosomes [154]. BMSC-derived exosomes could be taken up not only by MM cells but also by MDSCs in the BM and activate these MDSCs through STAT3 and STAT1 pathways. Moreover, these exosomes promoted the survival of MDSCs in the BM and enhanced their immunosuppressive functions, thus favoring MM progression [154].

Overall, exosomes secreted from MM cells and BM-derived cells potentially play important roles in the modification of the BM microenvironment and are involved in the maintenance of a vicious cycle between normal BM cells and MM cells (Figure 2). However, the cross talks between MM cells and other important BM-derived cells such as DCs, macrophages, and hematopoietic stem cells through EVs, especially exosomes, still need to be elucidated for a better understanding of the BM microenvironmental changes during MM progression.

### Potential therapeutic implications of EVs in MM

Despite substantial improvement in survival of MM patients over the past decade, innovative therapeutic strategies are still needed to prolong progression-free and overall survival and to potentially increase cure rate. Most innovations in drug therapy have been the result of a better understanding of MM biology leading to the recognition of new drug targets. We believe that knowledge on MM associated EVs will be a valuable asset to develop new therapies, since they seem to have a wide range of biological effects related to the BM micro-environment and anti-tumor immunity.

DC based vaccination in relapsed MM is the subject of several recent and ongoing clinical trials [155–161]. As mentioned above, exosomes are a potential candidate for use in these vaccination strategies. Tumor-derived exosomes have been shown to be immunogenic and contain targets for DC vaccination in lymphoma [162] and melanoma [163]. Furthermore dexosomes could in theory, be easier to handle than DCs, while retaining the same potential for tumor vaccination. This has been the subject of various clinical trials [164–166], but the induced immune responses seem to be dependent on additional factors creating either an immune stimulatory or an immune suppressive environment [167].

On the other hand, tumor-derived exosomes can be responsible for evading antibody-based therapy. It has been shown in lymphoma that CD20-carrying exosomes can bind the therapeutic anti-CD20-antibodies (Rituximab) [168] and thereby prevent lymphoma cell death by both antibody-dependent cell cytotoxicity (ADCC) and complement-dependent cytotoxicity (CDC). Already three hours after Rituximab infusion in lymphoma patients, one third to one half of the Rituximab present in the plasma was bound to these lymphoma-derived exosomes, and thus deemed ineffective [169]. The same protective effect was observed in breast cancer, where HER2 positive breast cancer-derived exosomes shield the tumor cells from the therapeutic effects of the anti-HER2 antibody Trastuzumab [170]. One could argue that MM-derived EVs might be responsible for a reduced effect of the new monoclonal antibody therapies eg. Daratumumab, Elotuzumab or Indatuximab Ravtansine in a similar way, by acting as a decoy for the antibodies.

Since tumor-derived exosomes can seemingly suppress the anti-tumor response and aid in a tumor-friendly microenvironment, research is ongoing on how to block the secretion of these exosomes, or eradicate them from the patient. Multiple inhibitors which block the release of EVs, such as GW4869, spiroepoxide, dimethyl amiloride, and manumycin-A, have been studied *in vitro* [171–173]. However these inhibitors are not specific and have too much off-target effects to be therapeutically practical. A better option might be to remove tumor-derived exosomes from the patient by using extracorporal hemofiltration [174].

### Perspectives

Although compelling studies have focused on the targets and functions of EVs secreted by many BM-derived cells and on the important role of EVs in the immune response, tumor progression, vaccine development, and treatment of various diseases, the complete crosstalk between these BM cells through EVs is still not fully elucidated. This is especially complex as the functions of EVs from many other BM cell types including endothelial cells, osteoclasts, osteoblasts, hematopoietic stem cells, NK cells, and lymphocytes are still not clear. Furthermore, other types of EVs such as oncosomes have not been studied yet in the BM microenvironment. Oncosomes are a subpopulation of membranous large MVs derived from cancer cells that are characterized by the ability to transfer oncogenic signals and protein complexes [175, 176]. The transfer of oncogene-containing oncosomes results in transformation-like changes in recipient cells [175, 177] and can induce phenotypic reprogramming of other normal cells [178, 179]. Therefore, further studies examining the role of EVs, including oncosomes in the BM crosstalk would help increase our understanding of the BM involvement in multiple disease types. Moreover, finding better ways to block pathological exosome secretion would benefit the development of novel therapies for cancers such as MM.
